# Age and site of Colonic Neoplastic Lesions: Implications of screening in South Asia

**DOI:** 10.12669/pjms.306.5701

**Published:** 2014

**Authors:** Manzoor Hussain, Abdullah Bin Khalid, Syed Ahsan, Wasim Jafri, Saeed Hamid, Anam Javed, Sana Wahab

**Affiliations:** 1 Manzoor Hussain, FCPS Medicine, Fellow Gastroenterology, Section of Gastroenterology, Department of Medicine, Aga Khan University Hospital, Karachi; 2 Abdullah bin Khalid, FCPS Medicine, FCPS Gastro, Section of Gastroenterology, Department of Medicine, Aga Khan University Hospital, Karachi, Pakistan.; 3 Syed Ahsan Ali, FCPS Medicine, Instructor in Dept. of Medicine, Pakistan. National Institute of Liver and Gastrointestinal Diseases, Dow University of Health Sciences, Karachi, Pakistan.; 4 SM Wasim Jafri, FRCP, FACG, Professor of Medicine, Section of Gastroenterology, Department of Medicine, Aga Khan University Hospital, Karachi, Pakistan.; 5 Saeed S Hamid. FRCP, FACG, Professor and Chair of Medicine, Section of Gastroenterology, Department of Medicine, Aga Khan University Hospital, Karachi, Pakistan.; 6 Anam Javed, Medical Student, Section of Gastroenterology, Department of Medicine, Aga Khan University Hospital, Karachi, Pakistan.; 7 Sana Wahab, Medical Student, National Institute of Liver and Gastrointestinal Diseases, Dow University of Health Sciences, Karachi, Pakistan. National Institute of Liver and Gastrointestinal Diseases, Dow University of Health Sciences, Karachi, Pakistan.

**Keywords:** Colonic Neoplastic Lesion, Colorectal Carcinoma

## Abstract

***Objective*** **:** To evaluate the Age of patients and the site of Colonic Neoplastic Lesions (CNL) and to determine the appropriate screening strategy for Colorectal Carcinoma (CRC) (sigmoidoscopy versus colonoscopy) in our population.

***Methods*** *:* This is a cross sectional study. Data of all patients more than 16 years of age who underwent full colonoscopic examination at the Aga Khan University hospital between January 2011 till December 2013 and were diagnosed to have CRC or advanced adenomas (defined as polyp more than 1 cm and/or having villous morphology on histology) was recorded. Lesions found distal to the splenic flexure were characterized as distal lesions and while lesions found between the splenic flexure and the cecum were characterized as proximal lesions.

***Results:*** During the study period colonic neoplastic lesions were found in 217 patients; 186 (85.7%) patients had CRC and 31(14.3%) patients had advanced adenomatous polyps. Mean age was 55.8±14 years and amongst them 72 (33.2%) patients were less than 50 years of age while 145 (66.8%) were more than 50 years. In 144 (66.4%) patients lesions were located in the distal colon, 65 (30%) had lesions in the proximal colon while in 8 (3.7%) patients the neoplastic lesions were found both in the proximal and distal colon. The predominant symptoms were bleeding per rectum in 39.6% of patients followed by weight loss in 31.8% of patients. Only 3 patients had familial syndromes with multiple polyps. When patients younger than 50 years of age were compared with patients more than 50 years there was no statistically significant difference between the site of neoplastic lesion as well as the presenting symptoms.

(p value 0.85).

***Conclusion:*** Colonic Neoplastic Lesions presented at younger age in our study population and one third of the lesions were found in the right sided colon. Hence screening for CNLs should be implied at an earlier age preferably with colonoscopy. More population based data is required to further validate our results.

## INTRODUCTION

Colorectal cancer (CRC) is a major health problem and is a leading cause of morbidity and mortality.^1^ It is predominantly a disease of middle age adults, with more than 90% of cases diagnosed in individuals older than 50 years.^2^^,^^3^ Compared with the West, CRC in South Asia has been reported to occur with a greater frequency in young patients.^4^^,^^5^ Data regarding colon cancer is limited in Pakistan.^5^ However in a recent study among patients less than 50 years of age a significant percentage of patients were reported to have CRC.^6^

CRC predominantly occurs in the left side of colon as seen in many of the studies in which the incidence of left sided CRC is more than the right sided^7^^,^^8^, however there are few European as well as regional studies that suggest the right shift of CRC.^6^^,^^9^^,^^10^ The change was due to a rise in the incidence of proximal lesions and a simultaneous fall in the incidence of distal lesions.^11^ These observed trends in cancer site distributions could reflect screening practices.^12^^,^^13^

Guidelines recommend screening for CRC at 50 years age group.^14^ The cutoff of 50 years is chosen because the incidence of CRC begins to rise at 50 years in Western population.^15^ Considering the fact that some of the recent reports from this region revealed that CRC begins to appear much earlier in this region and data from large scale population based studies is limited, it seems imperative to define the most appropriate age for screening for CRC.^16^

The endoscopic tools for screening are either sigmoidoscopy or colonoscopy. Sigmoidoscopy is easy to perform, has less patient discomfort and is able to detect lesions located distal to splenic flexure. Colonoscopy on the other hand requires more expertise to perform with increased discomfort and potential risk of complications, however can detect lesions anywhere in the colon.

Considering the high frequency of CRC in young patients with no existing screening programme in our region it therefore seems prudent to know the age of patient and site of colonic lesions in the population so that appropriate endoscopic screening modality i.e. Sigmoidoscopy or colonoscopy can be chosen for early detection and management of CRC.

## METHODS

***Operational definition: ***Colonic Neoplastic Lesion (CNL) constitutes advanced adenomas as well as CRC. Advanced adenomas comprise of adenomatous polyps greater than 1 cm, with villous histology (i.e. at least 25% villous), an edenoma with high grade dysplasia or invasive cancer.^17^

This cross-sectional study included data of all patients who underwent full colonoscopic examination at the Aga Khan University Hospital from January 1^st^ 2011 till December 31^st^ 2013 and were found to have colonic neoplastic lesions (CNL) was recorded. The participants in this study included patients ranging from 16 to 50 years of age. Colonoscopies were done under conscious sedation with either mepiridine i.e. pethidine and midazolam, or fentanyl. All colonoscopies were performed with Olympus CF AL videoscope (Olympus Corporation, Tokyo, Japan). Vitals of each patient were checked after every five minutes while the procedure was taking place including heart rate, oxygen saturation and blood pressure. After the procedure, they were noted after every fifteen minutes for a period of two hours. The findings on colonoscopy were noted keeping in mind the type and location of the lesion.

CNLs found distal to the splenic flexure were characterized as distal lesions and thus reachable with the help of sigmoidoscope, while CNLs found between the splenic flexure and the cecum were characterized as proximal lesions. All the samples were sent for histopathological diagnosis. 

After completion of the procedure, patients were followed up within two weeks in out-patient department and were managed as per biopsy results. This study was approved by the Ethical Review Committee of Aga Khan University Hospital.

***Statistical analysis: ***Data entry and the analysis were performed by using Statistical Packages for Social Sciences version 15 (SPSS, Chicago, Illinois, USA). Descriptive statistics were calculated for continuous variables such as age. Mean ± SD were computed. For categorical variables such as sex and type of lesion, the frequencies and percentages were calculated. Chi square test was applied for categorical variables for comparing the site of colonic neoplastic lesions i.e. proximal and distal between patients less than 50 versus patients more than 50 years of age. P < 0.05 was considered significant.

## RESULTS

During the study period 3,189 colonoscopies were performed. CNLs were found in 217 patients; 186 (85.7%) patients had CRC and 31 (14.3%) patients had advanced adenomas . Mean age was 55.8±14 years and amongst them 72 (33.2%) patients were less than 50 years of age while 145 (66.8%) were more than 50 years. The predominant symptoms were Bleeding per rectum in 86 (39.6%) of patients followed by Weight loss and Altered bowel habits 69 (31.8 %) and 36 (16.6%) respectively, Anemia without bleeding per rectum was seen in 22 (10.1%) patients and Abdominal pain in 4 (1.8%) patients. There were 22 (10.1%) patients who had two or more symptoms. In 144 (66.4%) patients lesions were located in the distal colon, 65 (30%) had lesions in the proximal colon while in 8 (3.7%) patients CNLs were found both in the proximal and distal colon (Table-I). Only 3 patients had familial syndromes with multiple polyps and the youngest amongst them was 16 years of age.

When patients younger than 50 years of age were compared with patients more than 50 years, there was no statistically significant difference between the site of neoplastic lesion as well as the presenting symptoms. (p value 0.85). Fig-I.

**Fig-I:**
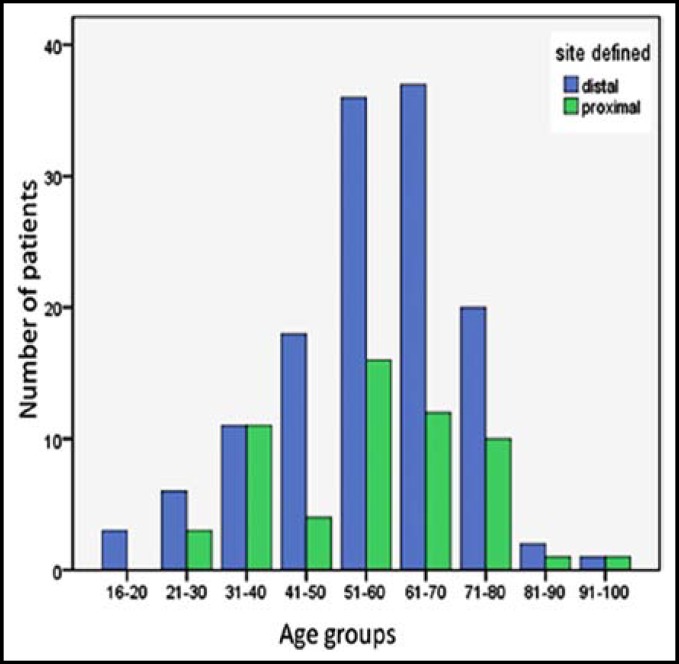
Number of distal and proximal CNL in relation to different age groups

**Table-I:** Distribution of Colonic Neoplastic Lesions (CNL) according to age and site

**Site of lesions n(%)**	**Patients less than 50 years n(%)**	**Patients more than 50 years n(%)**	**p value**
Distal lesions n= 144(66)	49(68)	95(66)	0.85
Proximal lesions n= 65(30)	21(29)	44(30)	0.82
Both Distal and Proximal lesions n = 08(4)	02(3)	06(4)	0.75
Total n= 217	72(33)	145(67)	

## DISCUSSION

This study was conducted to determine the implication of screening for CRC on the basis of age and site of CNLs. According to our findings the mean age of patients with CRC was 55.8±14 which is corresponding to local and regional studies^4^^-^^8^ and among them 72 (33%) patients were below 50 years of age. This is in contrast to Western data where CRC is negligible in the early ages^18^^,^^19^ and where the incidence of CRC begins to rise in the sixth decade of life.^15^ It was striking to note that only 3 patients with CRC had familial syndromes in our cohort while all the other patients had sporadic CRC. A similar study from a neighbouring country had similar results with low mean age of CRC.^4^ Moreover a study done on South Asians inhabiting in the United Kingdom revealed that they had CRC at a relatively younger age group as opposed to Caucasians in the same country.^20^^,^^21^ This shows that South Asian population may possess an increasing risk of CRC at an early age. A rather alarming finding of our study was that the number of advanced adenomas were much less than those of CRC but the data from countries which have proper Colon cancer screening programmes show a reverse trend.^22^ In our region there is no colon cancer screening programme and thereby it is likely that most patients present at symptomatic stages. This may be the reason that we found more CRC as compared to advanced adenomas.

Majority of the cohort in our study had bleeding per rectum (BPR) which has always been an alarming feature of possible underlying organic pathology in the colon.^16^ It is previously reported that BPR in the young age group can be diagnosed with sigmoidoscopy^16^; same holds true in this study in which 90% of the lesions in patients with BPR were in the left sided colon. Though most of the lesions 144 (66.4%) were located in the distal colon, however significant number 65 (30%) were also seen in the proximal colon and in few 8 (3.7%) patients CNLs were located both in the proximal and distal colon (Table-I). Similar results were confirmed in the studies from the West showing almost 1/3 of the cancers to be right sided.^9^^,^^23^

There are some recent local and regional reports which suggest proximal migration of CNLs where significant number of lesions is seen in right side of colon^7^^,^^12^ and we also found that almost one third of the lesions were located in the proximal colon hence sigmoidoscopy may miss lesions in the right sided colon.

When patients younger than 50 years of age were compared with patients more than 50 years, there was no statistically significant difference between the site of neoplastic lesion as well as their presenting symptoms. In younger population the proximal lesions were found in 21 (29%) patients where as in patients more than 50 years the corresponding lesions were seen in 44 (31%) patients.

Colorectal cancer is one of the few preventable cancers.^24^ Therefore the aim of health care authorities throughout the world is to imply screening tests at a pre symptomatic stage so that early detection could be done. Guidelines therefore recommend sigmoidoscopy or colonoscopy at the age of 50 years.^14^ Sigmoidoscopy beginning at the age of 50 years has shown a mortality benefit from CRC in the Western population.^25^ Though Sigmoidoscopy can pick up the distal lesions, however proximal lesions i.e. located between splenic flexure and cecum may be missed.

Large scale population based data of CRC is scarce in South Asia. However recent studies have shown that CRC presents at an earlier age in south Asian population than their Western counterparts^4^^,^^20^ and therefore there is a need to define the appropriate age for screening for CRC in South Asia.

***Limitation of the study:*** The major limitation of our study was that it was done on symptomatic patients but in the absence of population based data of colonoscopic findings, the results may be extrapolated to the general population.

## CONCLUSION

According to the study findings CNLs present at younger age in South Asian population and one third of the lesions were located in the right sided colon. Hence screening for CNLs should be implied at an earlier age preferably with colonoscopy. More population based data is required to further validate our results.

## Author’s Contribution:

**MH** conceived, designed, did statistical analysis and manuscript writing.

**ABK** involved in statistical analysis and manuscript editing.

**SA, AJ, SW** were involved in making questionnaire and data collection.

**WJ and SH** reviewed and approved the manuscript.
